# Klotho deficiency aggravates diabetes-induced podocyte injury due to DNA damage caused by mitochondrial dysfunction

**DOI:** 10.7150/ijms.49690

**Published:** 2020-09-28

**Authors:** Zhi Chen, Qing Zhou, Cong Liu, Yiping Zeng, Shaolong Yuan

**Affiliations:** 1 University-Town Clinic, 958 hospital of PLA Army, Chongqing, 400020, People's Republic of China.; 2 School of Military Preventive Medicine, Army Military Medical University, Chongqing, 400020, People's Republic of China.; 3 Center of Laboratory Medicine, Chongqing Prevention and Treatment Center for Occupational Disease, Chongqing, 400060, People's Republic of China.; 4 Department of orthopedics, Chongqing general hospital, University of Chinese Academy of Sciences, Chongqing, 400014, People's Republic of China.

**Keywords:** Klotho, diabetes, mitochondrial dysfunction (MtD), podocyte injury, DNA damage

## Abstract

Diabetic nephropathy (DN) is a progressive disease, the main pathogeny of which is podocyte injury inducing glomerular filtration barrier and proteinuria. The occurrence and development of DN could be partly attributed to the reactive oxygen species (ROS) generated by mitochondria. However, research on how mitochondrial dysfunction (MtD) ultimately causes DNA damage is poor. Here, we investigated the influence of Klotho deficiency on high glucose (HG)-induced DNA damage *in vivo* and* in vitro*. First, we found that the absence of Klotho aggravated diabetic phenotypes indicated by podocyte injury accompanied by elevated urea albumin creatinine ratio (UACR), creatinine and urea nitrogen. Then, we further confirmed that Klotho deficiency could significantly aggravate DNA damage by increasing 8-OHdG and reducing OGG1. Finally, we demonstrated Klotho deficiency may promote MtD to promote 8-OHdG-induced podocyte injury. Therefore, we came to a conclusion that Klotho deficiency may promote diabetes-induced podocytic MtD and aggravate 8-OHdG-induced DNA damage by affecting OOG1.

## Introduction

The incidence and prevalence of diabetes mellitus (DM) have grown significantly throughout the world due to the overall increase in type 2 diabetes (T2D). As one of the most frequent complications of DM, diabetic kidney disease (DKD) defined as a syndrome, is the most common metabolic disease in the world that is the leading cause of end-stage renal disease (ESRD) comprising about 40% of patients with chronic kidney disease (CKD) 1,2. The onset of clinically overt DKD is defined as persistent proteinuria and is most closely associated with podocytopathies that results from damage to the glomerular filtration barrier at the level of the highly differentiated glomerular podocyte cells 3-5. Despite the status quo, the factors that precipitate the development and progression of DKD remain to be fully elucidated. Moreover, the molecular mechanisms leading to proteinuria and podocyte effacement are poorly understood.

DM is characterized by the increased levels of ROS leading to high levels of adenosine triphosphate (ATP) 6. Nevertheless, mitochondria is the energy powerhouse of cell by ATP synthesis through oxidative phosphorylation (OXPHOS) and plays a key role in apoptosis. Mitochondria also attaches great importance to renal function, of which dysfunction is becoming increasingly recognized as contributing to renal glomerular and tubular diseases 7-11. Besides, tubular epithelial cells are rich in mitochondrion. Mitochondria dysfunction (MtD) and ROS-induced damage are also well reported in glomerular podocytes, as they are highly specialized and terminally differentiated epithelial cells 12. As limited replicative capability of regeneration, podocytes injury may represent the major mechanism of progressive renal damage. Remarkably, mitochondrial electron transport chain is identified as the major non-enzymatic source of diabetes-induced ROS in podocytes that are believed to cause the onset of albuminuria followed by progression to renal damage through podocytes depletion 12. MtD is usually accompanied by a reduction in the efficiency of the DNA repair capacity and antioxidant defense, consequently leading to the accumulation of cellular damage 13,14. DNA continually exposed to exogenous and endogenous stressors will lead to DNA breaks, damage or improperly repair of which can activate pro-apoptotic pathways, or induce cellular senescence 14-16. One of the major oxidative DNA-damage products is 8-hydroxydeoxyguanosine (8-OHdG) that could be repaired by base-excision repair (BER) system. 8-oxo-deoxyguanosine DNA glycosylase 1 (OGG1), an enzyme that is involved in the process, is associated with the DNA repair activity and decreases risk for some oxidative stress-related diseases 17,18. Cells have developed complex DNA damage signaling and repair mechanisms, that were collectively called DNA damage response (DDR) 15,16. DNA damage also plays an important role in the development of DM and its complications 19,20, but deep molecular mechanisms remains to be studied.

α-Klotho, also known as Klotho, which predominantly produced in renal tubular epithelial cells, regulates ageing-related processes existing in membrane-bound and soluble forms 21. Klotho may function as a humoral factor, can attenuate the development of hyperglycemia in mice challenged with DM 22. Whether Klotho participates in regulating DNA damage to protect from MtD-induced podocytes injury remains unknown. In this study, our data showed that Klotho deficiency may cause loss of regulation in OGG1 expression and promote the generation of ROS and DNA damage, which eventually aggravated MtD in diabetes-induced podocytes.

## Materials

### Animals

Male C57BL/6 mice were purchased from Beijing HFK Biologic Technology (Beijing, China); α-Klotho deficiency (*KL^+/-^*, C57BL/6) mice generously provided by Army Military Medical University (Chongqing, China), were generated by mating pairs of heterozygous Klotho mice (*KL^+/-^*) and their genotypes were verified by Mouse Direct PCR Kit (B40015, Bimake, Selleck). We used the following specific primers: wild-type, forward 5′-TTGTGGAGATTGGAAGTG GACGAAAGAG-3′ and reverse 5′-CTGGAC CCCCTG-AAGCTGGA-GTTAC-3′; Klotho mutant, forward 5′-TTGTGGAGATTGGAAGTGGACG AAAGAG-3′ and reverse 5′-CGCCCCGACCGGAGCTGAGAGTA-3′. These primers were expected to produce 815 bp (WT) and 419 bp (Klotho-deficient) amplification products. The PCR conditions were as follows: denaturation at 94°C for 5 min, 30 cycles of 94°C for 30 s, annealing at 60°C for 1 min, and extension at 72°C for 45 s, and a final extension at 72°C for 10 min. All *KL^+/-^*, or WT (C57BL/6) mice at 8 weeks of age were treated with a single daily intraperitoneal dose of 55mg/kg streptozotocin (STZ) for 1 week. Non-diabetic mice were treated with citrate buffer for 7 days, i.p. Mice were weekly monitored for body weight and blood glucose (Roche Glucose meter).

### Podocyte culture

Immortalized human podocytes (AB8/13) were gifted by Dr. Moin A. Saleem (University of Bristol, UK) and maintained in RPMI 1640 medium (Life Technologies, Grand Island, NY, USA) supplemented with 10% FBS, 100μg/ml penicillin,100μg/ml streptomycin. To propagate podocytes, the culture medium was supplemented with 10 U/mL human recombinant γ-interferon (Sigma, St. Louis, MO, USA) to enhance expression of the T antigen, and cells were cultivated at 33°C (permissive conditions). To induce differentiation, podocytes were cultured on type I collagen at 37°C without γ-interferon for at least 14 days. Podocytes were cultured in 6-well plates and then preincubated with 400 pM of recombinant human Klotho protein (R&D Systems, USA) for 48 h before high glucose (HG) induction 23. In parallel, podocytes incubated with mannitol (30 mM) for same time were taken as negative control.

### Histology

Mouse kidney tissues were immersion-fixed in 4% paraformaldehyde/phosphate-buffered saline and embedded in paraffin after tissue dehydration. The sections were cut (2 μm thickness), dewaxed in xylene, and rehydrated in decreasing concentrations of ethanol. Histological and morphometric analysis was carried out on paraffin sections cut on a rotation microtome (Microm) and stained with hematoxylin-eosin and periodic acid-schiff, respectively. The mesangial matrix expansion and PAS area were analyzed by Image Pro plus 6.0.

Immunohistochemical staining was conducted using the HRP DAB kit (ZLI-9017, Zsbio). Kidney tissues were deparaffinized and rehydrated. For antigen retrieval, sections were submerged in citrate buffer (pH 6.0) at 100°C for 30 min. After blocking endogenous peroxidase activity and nonreactive sites, primary rabbit monoclonal to WT1 (1:50; ab89901 Abcam), was incubated overnight at 4°C. Goat-anti rabbit immunoglobulins and diaminobenzidine tetrahydrochloride solution were used to detect antibody binding.

### Urine micro albuminuria

Albumin concentration in spot urine samples was measured with a commercially available competitive enzyme-linked immunosorbent assay following the instructions of the manufacturer (TP0100-1KT, Sigma, USA) and was normalized to urine creatinine.

### ELISA assay of serum Klotho

The blood samples from mice were collected and centrifuged for 10 minutes at 3000 rpm (4°C), and the serum Klotho was measured in duplicate using a mouse Klotho ELISA kit according to the manufacturer's protocol (Cusabio, Cologne, Germany).

### Detection of ROS production

The detection of intracellular ROS was used an ROS-sensitive fluorescent probe 2',7'-S-dichlorodihydrofluorescein diacetate (DCFH-DA, HY-D0940, MCE). Podocytes were loaded with the fluoroprobe DCFH-DA (5 µM) at 37°C for 40 minutes in 200 ml serum-free RPMI 1640. The fluorescent level was observed under an inverted fluorescence microscope, and the fluorescence intensity was measured at 480 nm excitation and 525nm emission with a microplate reader (Thermo Fisher Scientific, Pittsburgh, PA).

### Western blotting analyses

The proteins isolated from podocytes or kidney tissues was separated by SDS-PAGE and transferred to polyvinylidene difluoride membranes (EMD Millipore, Billerica, MA). The membranes were incubated at 4°C overnight with the following primary antibodies: rabbit polyclonal antibody to NPHS2(Podocin) (1:1000; ab50339 Abcam), rabbit monoclonal to WT1 (1:1000; ab89901 Abcam), rabbit monoclonal to Klotho(1:1000; ab232366 Abcam), mouse monoclonal to 8-OHdG (1:500; sc-393871 Santa Cruz), mouse monoclonal to Synaptopodin (1:500; sc-515842 Santa Cruz), rabbit monoclonal Bax2 (1:500; ab32503 Abcam), rabbit polyclonal to Nephrin (1:200; bs-4886R, Bioss), mouse monoclonal to Drp1 (1:1000, sc-271583 Santa Cruz), rabbit polyclonal to OGG1 (1:5000; bs-3687R, Bioss), rabbit polyclonal to Caspase-3 p17 subunit (1:500; bs-20364R), rabbit polyclonal to β-actin (1:5000; bs-0061R, Bioss) and rabbit monoclonal to GAPDH (1:5000, ab181602 Abcam). Then, the secondary antibody was goat anti-rabbit IgG(H+L)/HRP antibody (1:5000, bs-40295G-HRP Bioss) and goat anti-mouse IgG(H+L)/HRP antibody (1:5000, bs-40296G-HRP Bioss) were applied. The signals were developed with the ECL-Plus Western Blotting Detection System (GE Healthcare, Buckinghamshire, UK), and the densitometry analysis was performed with an image analysis system (Bio-Rad). The gray value was calculated by Image J. The ratio of the gray value of the target band to the internal reference band β-actin was taken as the relative expression level of protein. Each experiment was repeated three times.

### Immunofluorescence assay

The paraffin sections were de-paraffinized, antigen retrieval and incubated in goat serum for blocking. After incubation with the primary antibody (mouse monoclonal to 8-OHdG, 1:50; mouse monoclonal to Synaptopodin, 1:30; Rabbit polyclonal to Podocin, 1:100; rabbit polyclonal to Nephrin, 1:20; rabbit polyclonal to OGG1, 1:50) diluted in goat serum at 4°C overnight, the slides were incubated with the corresponding secondary antibody. Finally, all sections were counterstained with DAPI and sealed with Antifade Polyvinylpyrrolidone Mounting Medium (P0123, Beyotime). Immunofluorescent staining and images were obtained by a LSM780 laser scanning confocal microscope (ZEISS, Germany) system.

### Transmission electron microscopy (TEM)

TEM images were analyzed using Image Pro plus 6.0. The GBM thickness, foot process width and the number of foot processes per μm of GBM were calculated using a curvimeter (SAKURAI CO., LTD, Tokyo, Japan).

### Statistical analyses

Data are expressed as means ± SE. Student's *t*-test was employed for comparisons between two groups. Multiple comparisons were performed using one-way ANOVA, followed by Bonferroni's post hoc test. *P* values<0.05 were considered significant and are indicated in the figures by asterisks (*p<0.05; **p<0.01; ***p<0.001). Analyses were performed using Graph Pad Prism software (GraphPad Software Inc, version 7.0).

## Results

### Klotho deficiency exacerbated diabetic nephropathy

**Figure 1:**
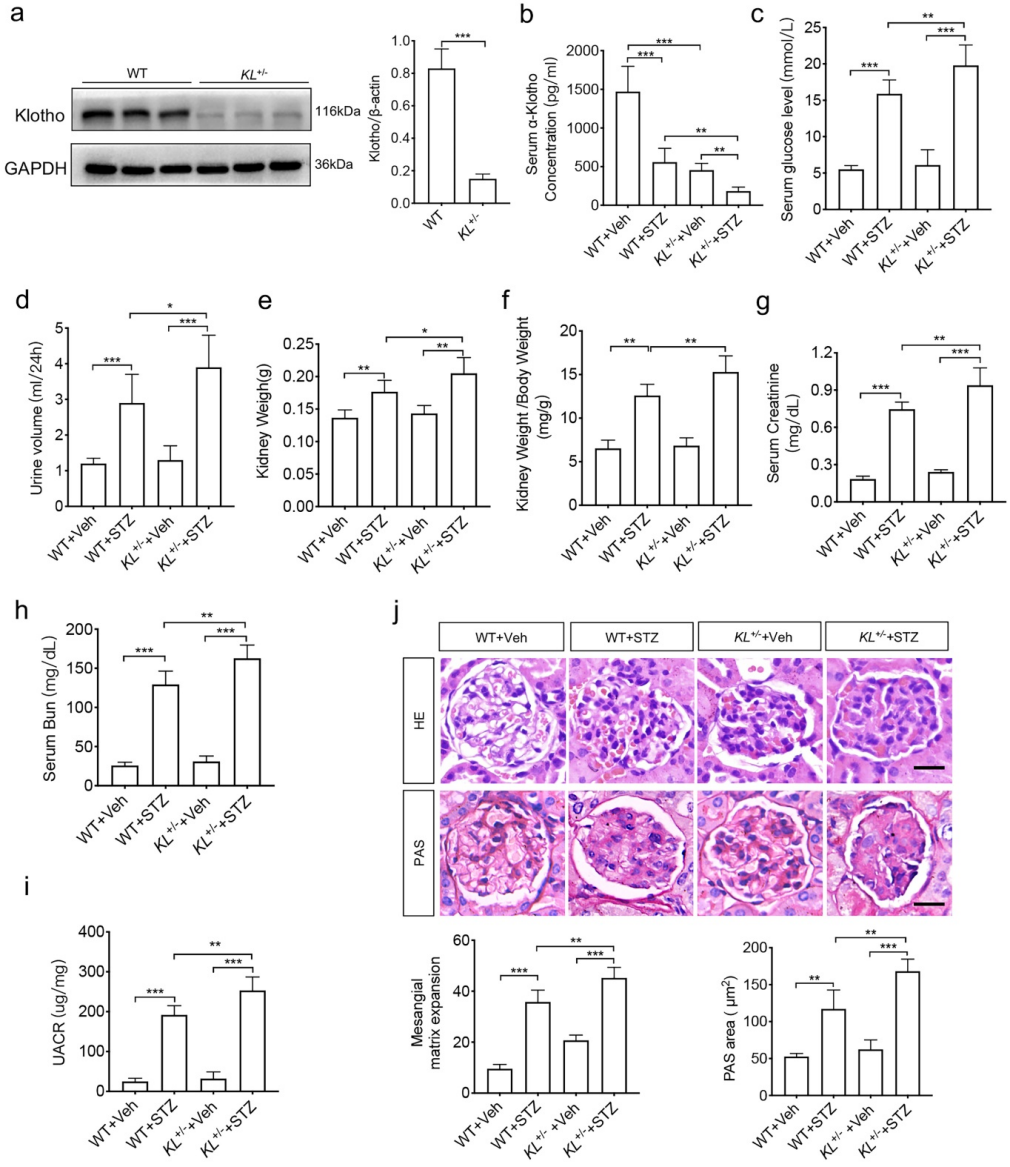
** Klotho deficiency may aggravate diabetes-induced renal dysfunction.** (a)The protein levels of Klotho in the kidneys were detected by immunoblotting. (b-e) Serum Klotho levels (Vehicle (injection of citrate buffer) =6, STZ (injection of 55 mg/kg/d STZ) =6) (b), serum glucose levels (c), Urine volume (d) and kidney weight (e). (f) Ratio of kidney weight to body weight in STZ-induced diabetic mice and normal mice. Comparison of serum creatinine (g) and Bun (h) between vehicle mice and STZ mice. (i)UACR (urine albumin-to-creatinine ratio) of STZ-induced diabetic mice and normal mice. (j) Comparison of mesangial matrix expansion and glycogen deposition in glomerulus of vehicle mice and STZ mice by HE staining and PAS staining, respectively. Scale bar: 20 µm. *P<0.05; **P<0.01; ***P<0.001. Veh, vehicle.

To analyze the relationship between Klotho and phenotypes of diabetic nephropathy, we carried out *in vivo* experiments. As high embryonic mortality and failure rate of diabetes model in *Klotho* homozygous mice, we selected *Klotho* heterozygous mice. To confirm the efficiency of Klotho depletion, the renal tissues from wild-type mice (WT) and Klotho deficiency mice (*KL*^+/-^) aged 8 to 10w (n=8/group) were subjected to western blot analysis. The results showed that Klotho significantly decreased in *KL*^+/-^ group (Fig. 1a). Then, we generated STZ-induced diabetic mice with WT or *KL*^+/-^ aged 8 to 10w (n=8/group). The concentration of Klotho in serum did decrease in diabetic mice, especially in STZ-induced *KL*^+/-^ mice (*KL*^+/-^ STZ) (Fig. 1b). Further compared with non-diabetic mice (n=8/group), all diabetic mice' concentration of blood sugar was higher than 13.8 mM and no downregulation was observed, and the levels further increased in *KL*^+/-^ STZ (Fig. 1c). Urine volume in diabetic WT group (WT STZ) was obviously higher than that of WT group and Klotho deficiency aggravated the phenomenon when compared to WT STZ (Fig. 1d). Diabetes also induced increase of kidney weight and ratio of kidney weight to body weight and Klotho deficiency induced greater increase of those (Fig. 1e and f). Meanwhile, we also measured serum creatinine, blood urea nitrogen (BUN) and UACR, respectively. We compared different groups and found the further rise of creatinine, BUN, as well as UACR in *KL*^+/-^ STZ when compared to WT STZ (Fig. 1g-i). To analyze the difference of glomerular morphology in different groups, we used hematoxylin-eosin staining and found mesangial matrix expansion in WT STZ. However, *KL*^+/-^ STZ appeared more mesangial matrix expansion as compared with WT STZ (Fig. 1j). Using periodic acid schiff (PAS) staining to observe glycogen deposition, we found there were glycogen deposition in glomerular of both WT STZ and *KL*^+/-^ STZ. Moreover, compared with WT STZ, glomerular accumulated more glycogen in *KL*^+/-^ STZ (Fig. 1j). All these data indicated Klotho deficiency may aggravate diabetic renal dysfunction.

### Klotho deficiency exacerbated STZ-induced podocyte injury

**Figure 2:**
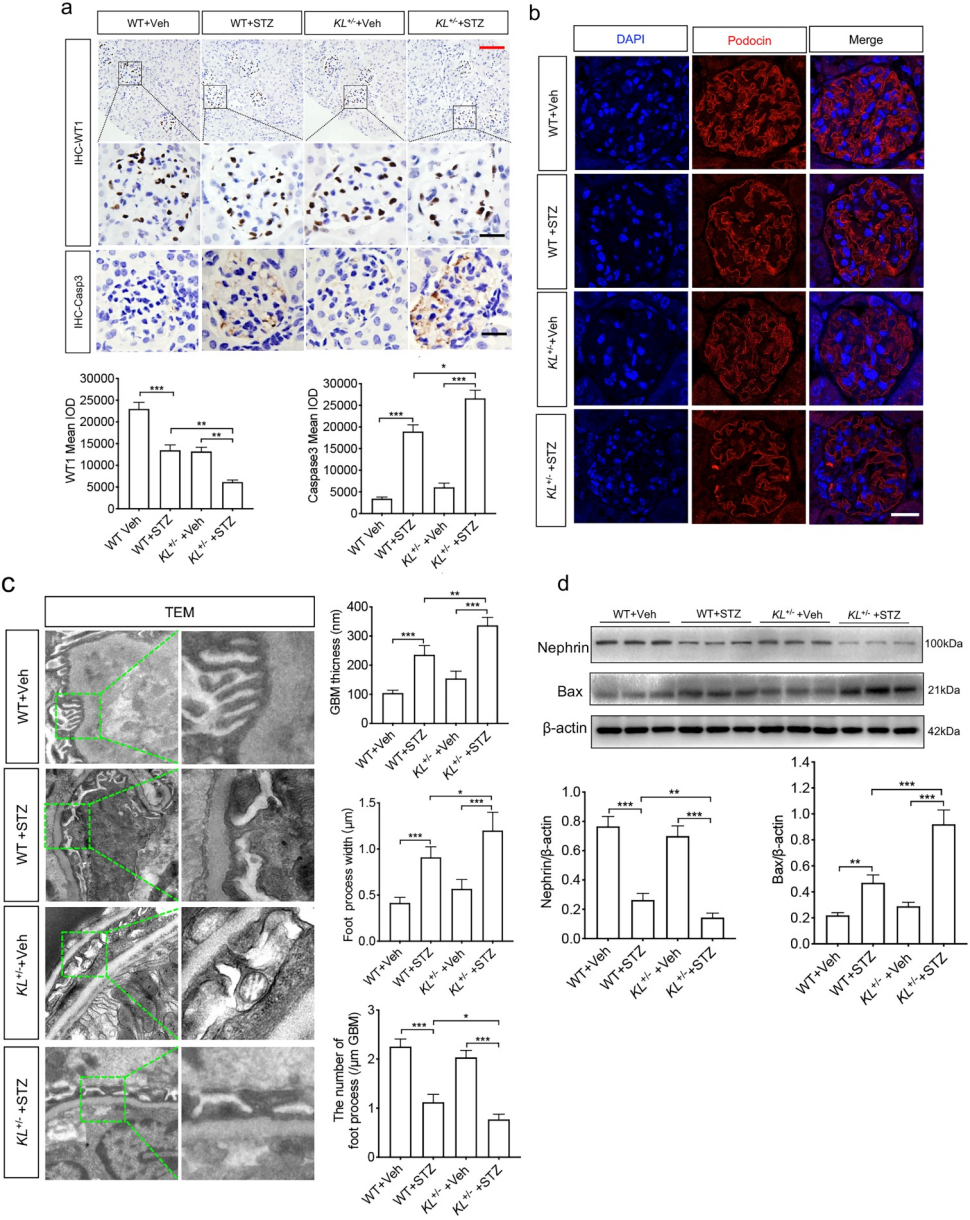
Klotho deficiency (*KL^+/-^*) promoted podocyte injury in STZ-induced mice. (a) Immunohistochemistry staining showing the expression of podocyte nuclear marker WT1 and Caspase-3 p17 subunit related to apoptosis in WT Vehicle, WT STZ, *KL^+/-^* Vehicle and *KL^+/-^* STZ. Scale bar, red 50 µm, black 20 µm. (b) Immunofluorescence staining showing the expression of podocyte membrane marker Nephrin in WT Vehicle, WT STZ, *KL^+/-^* Vehicle and *KL^+/-^* STZ. Scale bar, white 20 µm. (c) Representative photomicrographs and quantifications of mean glomerular basement membrane (GBM) thickness (yellow arrow), mean foot process width (red arrow), and the number of foot processes in different groups of mice by transmission electron microscopy (TEM) analyses. Scale bar, 200 nm. (d) Representative western blot gel documents and summarized data showing the relative protein level of Nephrin, PRDX2 and Bax in the kidney from WT Vehicle, WT STZ, *KL^+/-^* Vehicle and *KL^+/-^* STZ. *P<0.05; **P<0.01; ***P<0.001.

Whether Klotho deficiency induced podocyte injury, we examined the expression of WT1, podocyte's marker, and Caspase-3 known as apoptosis-related protein by immunohistochemistry (IHC). We found diabetes inhibited the expression of WT1 (Fig. 2a) and promoted expression of Caspase-3 (Fig. 2a). It's worth noting that Klotho deficiency pushed the pathological process (Fig. 2a). Podocytes are an integral part of the glomerular filtration barrier and Podocin (NPHS2), a podocyte split membrane protein, has major regulatory functions in the renal permeability of proteins, as well as Nephrin (NPHS1). Therefore, we further confirmed Podocin expression by immunofluorescence (Fig. 2b). Transmission electron microscope (TEM) suggested that podocyte was injury as evidenced by glomerular basement membrane (GBM) thickening, podocyte foot process broadening or effacement in WT STZ, that were significantly lower than *KL*^+/-^ STZ (Fig. 2c). Nephrin, another podocyte's marker, and apoptosis regulator Bax, acting as a role in mitochondrial apoptotic process, were also subjected to western blot analysis. We found Klotho deficiency further inhibited the expression of Nephrin and partially suppressed Bax in *KL*^+/-^ STZ as compared to WT STZ (Fig. 2d). The results suggested Klotho deficiency further exacerbated STZ-induced podocyte injury.

### Klotho deficiency aggravated HG-induced MtD of podocytes

**Figure 3:**
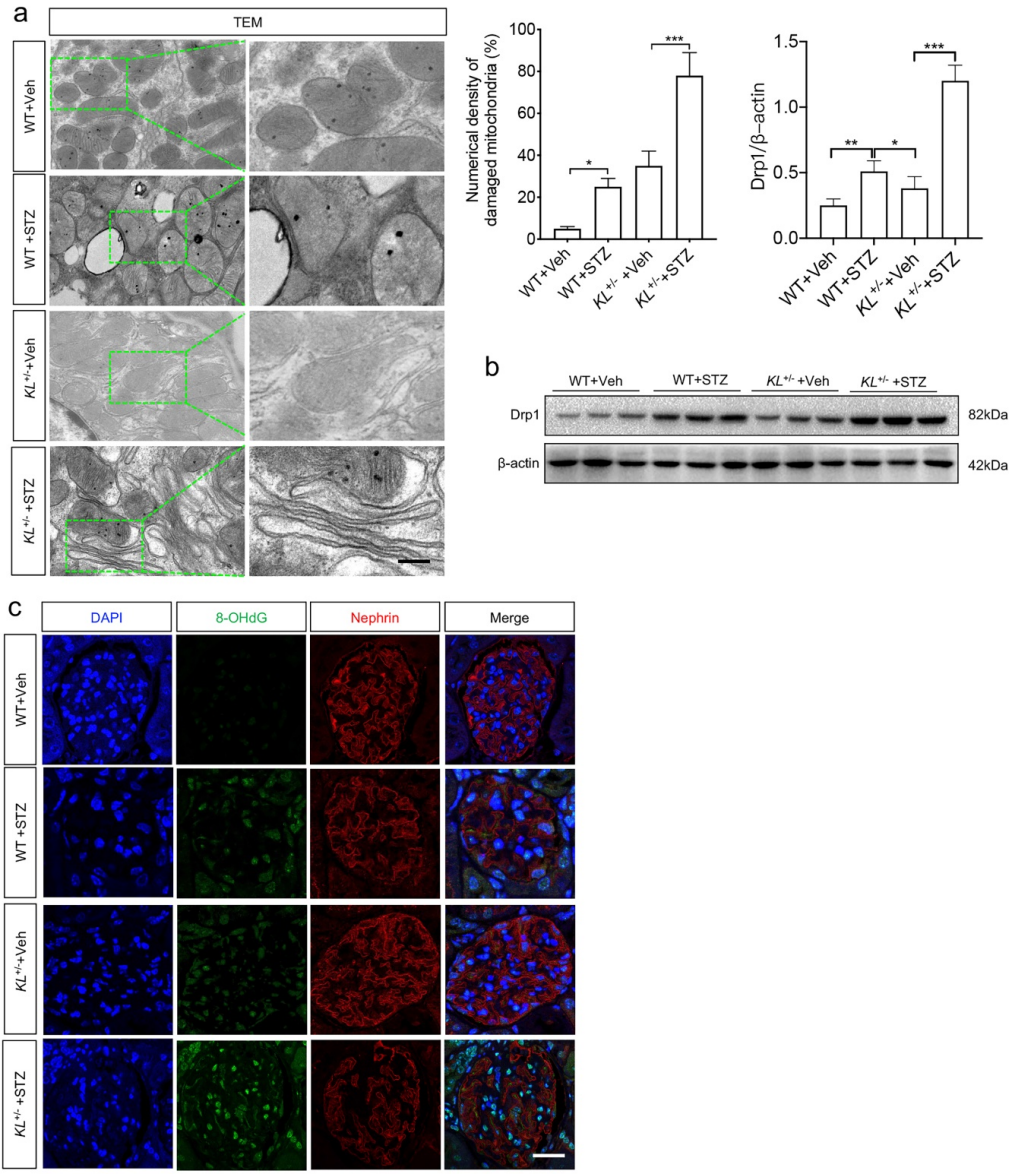
The effects of Klotho deficiency on mitochondrial morphology in diabetes-induced podocytes. (a) Changes of mitochondrial morphology in podocytes of each group. (b) Representative western blot gel documents and summarized data showing the expression of mitochondrial fission-related protein level of Drp1 in the kidney from WT Vehicle, WT STZ, *KL^+/-^* Vehicle and *KL^+/-^* STZ. *P<0.05; **P<0.01; ***P<0.001. (c) Immunofluorescence staining showing the expression of DNA damage marker 8-OhdG as well as Nephrin in WT Vehicle, WT STZ, *KL*^+/-^ Vehicle and* KL*^+/-^ STZ. Scale bar, white 20 µm.

As glucose metabolism is related to energy metabolism, mitochondria play an important role in this process. To wonder whether Klotho deficiency affected MtD of podocytes, TEM was performed again. The results showed mitochondrial hypertrophy appeared in podocytes of WT STZ when compared to that of WT (Fig. 3a). Meanwhile, numerical density of damaged mitochondria increased significantly in WT STZ as compared to WT Veh (Fig. 3a). However, slight mitochondrial contraction was observed in non-diabetic *KL*^+/-^ mice but this phenomenon was obviously aggravated in *KL*^+/-^ STZ (Fig. 3a). Moreover, higher numerical density of damaged mitochondria was observed in *KL*^+/-^ STZ when compared to WT STZ (Fig. 3a). We also explored the expression of Dynamin-related protein 1 (Drp1), the master regulator of mitochondrial fission. We observed the expression of Drp1 was significantly increased in WT STZ and further upregulated in *KL*^+/-^ STZ (Fig. 3b). Further analysis by immunofluorescence suggested 8-hydroxydeoxyguanosine (8-OHdG), a marker of DNA damage, increased with diabetes, especially in *KL*^+/-^ STZ, which was inversely related to the expression of Nephrin (Fig. 3b). The results indicated Klotho deficiency may be related to MtD of podocyte.

### Klotho deficiency affected OGG1 in HG-treated podocytes

**Figure 4:**
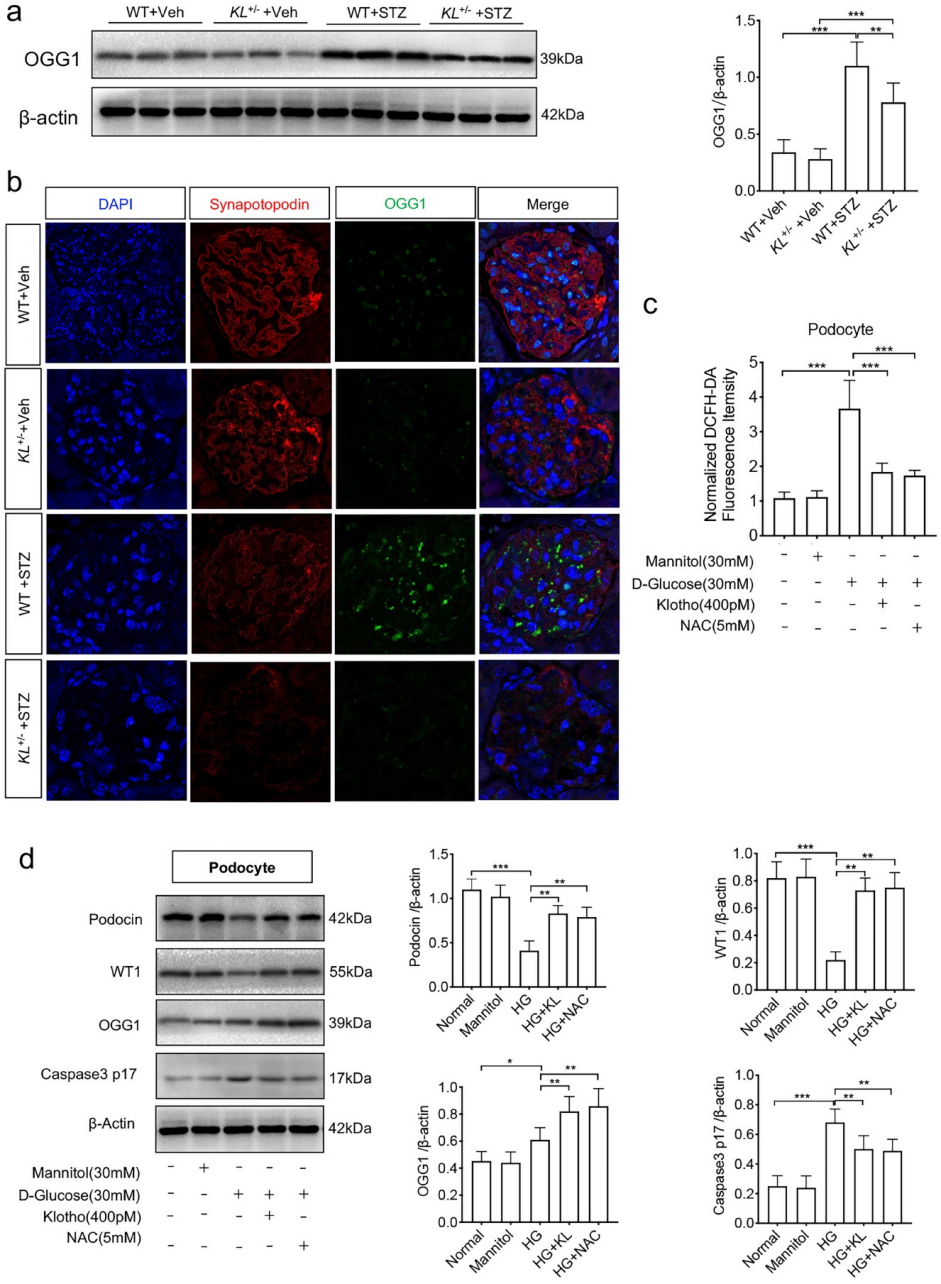
Klotho deficiency inhibited OGG1 aggravating podocytes injury in STZ-induced mice. (a) Representative western and summarized data showing the relative protein levels of OGG1 in the kidney from WT Vehicle, WT STZ, *KL^+/-^* Vehicle and *KL^+/-^* STZ. (b) Immunofluorescence staining showing the expression of OGG1 as well as podocyte membrane marker Synapotopodin in WT Vehicle, WT STZ, *KL^+/-^* Vehicle and *KL^+/-^* STZ. Scale bar, 20 µm. (c) Effect of Klotho on ROS generation in HG-induced podocytes. (d) Representative western blot gel documents and summarized data showing the relative protein level of Podocin, WT1, OGG1 and Caspase-3 p17 subunit in podocytes. *P<0.05; **P<0.01; ***P<0.001.

Having found the relationship between Klotho and 8-OHdG, we wonder whether DNA damage repair enzymes were suffered interference. We first detected 8-oxoguanine DNA glycosylase (OGG1), a DNA repair enzyme, by westernblot and found the Klotho deficiency was accompanied with low expression of OGG1 in diabetic mice (Fig. 4a). Further experimental confirmation by immunofluorescence showed STZ-induced diabetes suppressed the expression of both synapotopodin, a marker of podocyte, and OGG1. Klotho deficiency further inhibited the expression (Fig. 4b). Meanwhile, we analyzed the role of ROS in podocytes when treated with HG. We found a sharp rise of ROS in HG-induced podocytes (Fig. 4c). When applied with human Klotho recombinant protein or N-acetyl-L-cysteine (NAC), a ROS scavenger, HG-treated ROS was ameliorated, respectively (Fig. 4c). We also demonstrated the expression of WT1, Podocin, OGG1 as well as Caspse-3, respectively. Both Klotho and NAC could inhibit the reduction of WT1 and Podocin and inhibit the activation of Caspase-3 (Caspase p17) in HG-treated podocytes (Fig. 4d). Moreover, OGG1 was ameliorated by Klotho or NAC (Fig. 4d). The above results indicated that HG activated OGG1 which was disturbed by Klotho deficiency leading podocyte injury.

## Discussion

Diabetic nephropathy (DN) is a progressive disease that affects about one third of diabetic patients, the main pathogeny of which is podocyte injury that leads to glomerular filtration barrier and proteinuria 24,25. Klotho, anti-aging protein with critical roles in protecting kidney, is expressed predominantly in the kidney and secreted in the blood. Nonetheless, the mechanisms of Klotho ameliorating podocyte injury induced by ROS are of insufficient coverage. ROS mainly generates in mitochondria, of which dysfunctions have been associated with apoptosis, aging, and a number of pathological conditions 26. Here, we investigated the function of Klotho deficiency on MtD of diabetes-induced podocytes. We found Klotho deficiency aggravated HG-induced MtD and podocyte injury resulting in renal dysfunction *in vitro* and *in vivo* experiments. Further experiments first showed Klotho deficiency promoted 8-OHdG to induce DNA damage by affecting OGG1 expression in HG-treated podocytes.

Podocyte is nonrenewable and vulnerable to a variety of injuries as is highly specialized, terminally differentiated epithelial cells that line the outer surface of the glomerular basement membrane 27-29. The correlation of research between Klotho and ROS in diabetes is mainly in ROS-induced podocyte apoptosis 12. Enhanced production of ROS has been recognized as the major determinant of age-related endothelial dysfunction 30,31. p66SHC, a redox enzyme that generates mitochondrial ROS (hydrogen peroxide) as signaling molecules for apoptosis, induced by HG could mediate mitochondrial dysfunction 12,32,33. Based on previously study, we confirmed that HG induced the production of ROS and MtD in podocytes. Furthermore, Klotho deficiency actually aggravated HG-induced ROS and MtD which eventually deteriorated diabetic nephropathy. Moreover, our study focused on diabetes-induced DNA damage and found the relationship between Klotho and DNA damage in diabetes nephropathy (DN). We found Klotho deficiency triggered 8-OHdG-induced DNA damage and inhibited OGG1 expression in HG-treated podocytes.

A recent report demonstrated that Klotho could act as a versatile hormonal factor to protect cells from oxidation and cellular apoptosis by reducing tacrolimus-induced mitochondrial dysfunction 34. Previous reports also have confirmed an impaired mitochondrial morphology with decreased α-Klotho levels in Klotho knockout mice 35,36. However, the mechanism of regulating mitochondrial function by Klotho has remained poorly understood. It is difficult to discern whether the MtD is a primary result of Klotho deficiency. Noteworthily, our study found Klotho deficiency may aggravate podocyte MtD for the failure of regulating ROS-induced low expression of OGG1 and deteriorate 8-OHdG-induced DNA damage in diabetes. Klotho also plays an important role in many degenerative diseases such as chronic renal failure (CRF), osteoporosis and arteriosclerosis 37, 38. In diabetic nephropathy, Klotho is also reported to protect from glomerular hypertrophy in a cell cycle-dependent manner. Our results further confirmed the protective roles of Klotho on diabetes-induced DNA damage of podocyte, how Klotho regulates OGG1 inhibiting 8-OHdG remains to be further studied. OGG1 is the major DNA glycosylase in human cells for removing 8-OHdG, one of the most frequent endogenous base lesions formed in the DNA of aerobic organisms. OGG1 could be phosphorylated *in vivo* on a serine residue and is subject to protein kinase C (PKC)-mediated phosphorylation *in vitro*, suggesting that PKC is responsible for the phosphorylation event 39. Nevertheless, Protein kinase C (PKC) transducing signals is mediated by diacylglycerol (DG) or the second messengers Calcium ion 40. Klotho as a regulator of calcium homeostasis inhibits transient receptor potential channel 6 (TRPC6)-mediated Ca^2+^ influx in cultured mouse podocytes and ameliorates albuminuria and renal fibrosis 41,42. In summary, Klotho may be as a regulator of calcium to inhibit PKC and then activate OOG1, but further molecular mechanisms need further study.

## Conclusions

Our results imply that Klotho deficiency may aggravate podocyte MtD by affecting ROS and 8-OHdG-induced DNA damage by affecting OOG1 in diabetes. The mechanism may be the deeper cause of podocyte injury as subjected to HG.
